# Muscle strength and muscle mass as predictors of hospital length of stay in patients with moderate to severe COVID‐19: a prospective observational study

**DOI:** 10.1002/jcsm.12789

**Published:** 2021-09-14

**Authors:** Saulo Gil, Wilson Jacob Filho, Samuel Katsuyuki Shinjo, Eduardo Ferriolli, Alexandre Leopold Busse, Thiago Junqueira Avelino‐Silva, Igor Longobardi, Gersiel Nascimento de Oliveira Júnior, Paul Swinton, Bruno Gualano, Hamilton Roschel, The HCFMUSP COVID‐19 Study Group

**Affiliations:** ^1^ Applied Physiology & Nutrition Research Group, School of Physical Education and Sport, Rheumatology Division, Faculdade de Medicina FMUSP Universidade de Sao Paulo São Paulo SP Brazil; ^2^ Laboratorio de Investigacao Medica em Envelhecimento (LIM‐66), Servico de Geriatria, Hospital das Clinicas HCFMUSP, Faculdade de Medicina Universidade de Sao Paulo Sao Paulo Brazil; ^3^ Rheumatology Division, Hospital das Clinicas HCFMUSP, Faculdade de Medicina FMUSP Universidade de Sao Paulo Sao Paulo Brazil; ^4^ Division of Internal and Geriatric Medicine, Department of Internal Medicine – Ribeirão Preto Medical School Universidade de Sao Paulo Ribeirao Preto Brazil; ^5^ School of Health Sciences Robert Gordon University Aberdeen UK

**Keywords:** COVID‐19, Handgrip, Hospital stay, Skeletal muscle

## Abstract

**Background:**

Strength and muscle mass are predictors of relevant clinical outcomes in critically ill patients, but in hospitalized patients with COVID‐19, it remains to be determined. In this prospective observational study, we investigated whether muscle strength or muscle mass are predictive of hospital length of stay (LOS) in patients with moderate to severe COVID‐19 patients.

**Methods:**

We evaluated prospectively 196 patients at hospital admission for muscle mass and strength. Ten patients did not test positive for SARS‐CoV‐2 during hospitalization and were excluded from the analyses.

**Results:**

The sample comprised patients of both sexes (50% male) with a mean age (SD) of 59 (±15) years, body mass index of 29.5 (±6.9) kg/m^2^. The prevalence of current smoking patients was 24.7%, and more prevalent coexisting conditions were hypertension (67.7%), obesity (40.9%), and type 2 diabetes (36.0%). Mean (SD) LOS was 8.6 days (7.7); 17.0% of the patients required intensive care; 3.8% used invasive mechanical ventilation; and 6.6% died during the hospitalization period. The crude hazard ratio (HR) for LOS was greatest for handgrip strength comparing the strongest versus other patients (1.47 [95% CI: 1.07–2.03; *P* = 0.019]). Evidence of an association between increased handgrip strength and shorter hospital stay was also identified when handgrip strength was standardized according to the sex‐specific mean and standard deviation (1.23 [95% CI: 1.06–1.43; *P* = 0.007]). Mean LOS was shorter for the strongest patients (7.5 ± 6.1 days) versus others (9.2 ± 8.4 days). Evidence of associations were also present for vastus lateralis cross‐sectional area. The crude HR identified shorter hospital stay for patients with greater sex‐specific standardized values (1.20 [95% CI: 1.03–1.39; *P* = 0.016]). Evidence was also obtained associating longer hospital stays for patients with the lowest values for vastus lateralis cross‐sectional area (0.63 [95% CI: 0.46–0.88; *P* = 0.006). Mean LOS for the patients with the lowest muscle cross‐sectional area was longer (10.8 ± 8.8 days) versus others (7.7 ± 7.2 days). The magnitude of associations for handgrip strength and vastus lateralis cross‐sectional area remained consistent and statistically significant after adjusting for other covariates.

**Conclusions:**

Muscle strength and mass assessed upon hospital admission are predictors of LOS in patients with moderate to severe COVID‐19, which stresses the value of muscle health in prognosis of this disease.

## Introduction

Aging and chronic conditions such as type 2 diabetes increase the risk of developing severe forms of COVID‐19. Nevertheless, apparently healthier, younger individuals may also require hospitalization and develop poor outcomes.^1^, ^2^, ^3^ This suggests that there might be undiscovered clinical features associated with COVID‐19 prognosis, with muscular parameters being potential candidates.

Skeletal muscle constitutes ~40% of total body mass and plays a pivotal role in different physiological process such as immune response, regulation of glucose levels, protein synthesis, and basal metabolic rate.^4^, ^5^, ^6^ Handgrip strength and muscle mass have been shown previously to be predictive of clinical outcomes, such as hospital length of stay (LOS) and mortality, in distinct populations.^7^, ^8^, ^9^, ^10^, ^11^

In fact, the significance of muscle mass and strength to exercise and activities of daily living has never been questioned. Muscle also plays a central role in the response to stress in acute conditions, a role that has been somewhat less appreciated.^12^ Cases of exacerbated cytokines production (i.e. ‘cytokine storm’) can lead to sepsis, which has been deemed partially responsible for fatal cases of COVID‐19.^13^ In such conditions, inflammation leads to multi‐organ damage, affecting mostly pulmonary, cardiac, hepatic, vascular, and renal systems. Preservation of protein content in key tissues and organs, such as the brain, heart, and liver, is essential for survival, and it can be maintained relatively constant under acute stressful conditions, provided muscle mass is adequate to supply the required amino acids.^14^, ^15^ Skeletal muscle is also a major immunoregulatory organ, responsible for the production of a wide range of soluble factors with anti‐inflammatory and immunoprotective effects, the so‐called myokines, which could help ameliorate exacerbated inflammation in this disease.^5^ The potentially protective role of muscle tissue in COVID‐19 allows hypothesizing that muscle health may be an important predictor of clinical outcomes in this disease.

Herein, we investigated whether muscle strength and muscle mass assessed at hospital admission are predictive of LOS in patients with moderate to severe COVID‐19.

## Methods

### Study design

This is a prospective observational study conducted between March 2020 and October 2020 in the Clinical Hospital of the School of Medicine of the University of Sao Paulo in Brazil (HCFMUSP) the largest quaternary referral teaching hospital in Latin America. This study was approved by the local Ethics Committee (Ethics Committee Approval Number (31303720.7.0000.0068). All patients provided written informed consent before entering the study. This manuscript was reported according to the Strengthening the Reporting of Observational Studies in Epidemiology (STROBE) Statement and comply with ethical guidelines for publishing in the *Journal of Cachexia, Sarcopenia and Muscle*.^16^

### Participants

The inclusion criteria were (i) aged 18 or older; (ii) diagnosis of COVID‐19 by PCR for SARS‐CoV‐2 from nasopharyngeal swabs or computed tomography scan findings (bilateral multifocal ground‐glass opacities ≥50%) compatible with the disease; (iii) diagnosis of flu syndrome with hospitalization criteria on hospital admission, presenting respiratory rate ≥24 breaths per minute, saturation <93% on room air or risk factors for complications, such as heart disease, diabetes mellitus, systemic arterial hypertension, neoplasms, immunosuppression, pulmonary tuberculosis, and obesity, followed by COVID‐19 confirmation. Exclusion criteria were (i) cancer in the past 5 years; (ii) delirium; (iii) cognitive deficit that precluded the patient from reading and signing the informed consent form; (iv) prior diagnosis of muscle degenerative disease (e.g. myopathies, amyotrophic lateral sclerosis, and stroke); (v) patients already admitted under invasive mechanical ventilation. Patients who met these criteria were considered to have moderate to severe COVID‐19 according to NIH.^17^ Patients or the public were not involved in the design, or conduct, or reporting, or dissemination plans of our research.

### Data collection

All patients were evaluated in the point‐of‐care within <48 h upon hospital admission for handgrip strength and vastus lateralis cross‐sectional area, by means of ultrasound imaging, and were followed until medical discharge.

Handgrip strength assessments were performed with the patient seated holding the dynamometer (TKK 5101; Takei, Tokyo, Japan) with the dominant hand and elbow positioned at a 90° angle. Three maximum attempts of 5 s with 1 min of the interval between attempts were performed, and the best result was used for analysis. Vastus lateralis cross‐sectional area was assessed by a B‐mode ultrasound with a 7.5‐MHz linear‐array probe (SonoAce R3, Samsung‐Medison, Gangwon‐do, South Korea) as previously described.^18^ Cross‐sectional area analyses were performed in a blinded fashion by a single investigator using ImageJ (NIH, USA). All tests were conducted by the same investigator to avoid bias. Coefficients of variation for handgrip strength and vastus lateralis cross‐sectional area were 4.1% and 3.5%, respectively. Demographic, clinical, and biochemical data of the patients were obtained through medical records.

### Outcome and stratification of patients

Our primary outcome was LOS, defined as time (days) from hospital admission to medical discharge. To examine whether muscle strength or mass were predictive of LOS, we ranked patients according to handgrip strength and vastus lateralis cross‐sectional area into sex‐specific tertiles. Then, we compared the highest tertile (High) versus the combined mid and lowest tertiles (High vs. Other), and the lowest tertile (Low) versus the combined mid and highest tertiles (Low vs. Other).

### Sample size and statistical analyses

An *a priori* sample size estimate was made to achieve small optimism in the predictor effect estimates as defined by a global shrinkage factor of 0.9.^19^ The expected shrinkage is conditioned on the sample size (*n*), the total number of predictors (p) and a generalization of the proportion of variance explained for multivariable models with time‐to‐event outcomes ( $R_{\mathrm{CS}\text{\_}\mathrm{app}}^{2}$).^19^ For the present study, p was set to 6 and $R_{\mathrm{CS}\text{\_}\mathrm{app}}^{2}$ to 0.5 based on findings from previous research^8^, ^20^, ^21^ and therein, indicating a required sample size of *n* = 184. Guided by this estimate, a total of 196 patients were evaluated during their hospital stay.

The outcome (LOS) was analysed with multivariable Cox proportional baseline hazard models with surviving patients only and adjusted for sex (male or female), age group (18–35, 36–55, or ≥56), obesity (BMI < 30 or BMI ≥ 30), oxygen support at admission (0–4 L, 5–9 L,and ≥10 L), and Type 2 diabetes (yes or no). Both crude and adjusted hazard ratios (HRs) were estimated for handgrip strength and vastus lateralis cross‐sectional area. Each predictor was assessed as both a discrete and continuous predictor. Discrete models were conducted by calculating sex‐specific tertiles as originally planned and then focusing on either the largest tertile (High vs. Other) or the smallest tertile (Low vs. Other). Continuous models were also included and conducted by standardizing predictor values relative to the sex‐specific mean and standard deviation. HRs were accompanied with corresponding 95% confidence intervals (95% CI), with all analyses performed in the statistical environment R (version 3.5.3; R Core Team 2020) with the survival^22^ and survminer^23^ packages.

## Results

### Patients

One hundred ninety‐six patients were evaluated. Ten patients did not test positive for SARS‐CoV‐2 during the hospitalization period and were excluded from the analyses. *Table* 1 shows the demographic, biochemical, and clinical characteristics of the patients. Overall, 86% (160 of 186) had a positive PCR test for SARS‐CoV‐2 at the enrollment, and 42% had computed tomography scan findings suggestive (i.e. pulmonary commitment ≥50%) for COVID‐19. All the remaining patients (26 of 186) had the diagnosis confirmed by serology assay (ELISA) to detect IgG against SARS‐CoV‐2 at some point during the hospital stay. The sample comprised patients of both sexes (50% male) with a mean (SD) age of 59 years (±15) and a body mass index of 29.5 kg/m^2^ (±6.9). The prevalence of current smoking patients was 24.7%, and more prevalent coexisting conditions were hypertension (67.7%), obesity (40.9%), and type 2 diabetes (36.0%).

**Table 1:** Demographics and clinical characteristics of patients at hospital admission

	All patients (*n* = 186)	Survivors (*n* = 174)
Sex, *n* (%)		
Female	93 (50.0%)	88 (50.6%)
Male	93 (50.0%)	86 (49.4%)
Age, *n* (%)		
<65	116 (62.4%)	112 (64.4%)
>65	70 (37.6%)	62 (35.6%)
Race, *n* (%)		
White	95 (51.1%)	90 (51.7%)
Black	58 (31.2%)	52 (29.9%)
Yellow	33 (17.7%)	32 (18.4%)
Smoking status, *n* (%)		
Never	140 (75.3%)	132 (75.9%)
Current	46 (24.7%)	42 (24.1%)
Co‐morbidities, *n* (%)		
Asthma	12 (6.5%)	11 (6.3%)
Heart failure	18 (9.7%)	16 (9.2%)
Obesity (BMI > 30)	76 (40.9%)	70 (40.2%)
Type 2 diabetes	67 (36.0%)	56 (32.2%)
COPD	7 (3.8%)	7 (4.0%)
Chronic renal disease	11 (5.9%)	9 (5.2%)
Ethilism	7 (3.8%)	6 (3.4%)
Atrial fibrillation	8 (4.3%)	8 (4.6%)
Acute myocardial infarction	13 (7.0%)	13 (7.5%)
Hypertension	126 (67.7%)	113 (64.9%)
COVID‐19 testing, *n* (%)		
Positive PCR for SARS‐CoV‐2	160 (86.0%)	148 (85.1%)
Positive IgG for SARS‐CoV‐2	26 (14.0%)	26 (14.9%)
Acute COVID‐19 symptoms, *n* (%)		
Anosmia	37 (19.9%)	35 (20.1%)
Headache	41 (22.0%)	38 (21.8%)
Runny nose	17 (9.1%)	17 (9.8%)
Diarrhoea	33 (17.7%)	31 (17.8%)
Dysgeusia	31 (17.2%)	30 (17.2%)
Dyspnoea	151 (81.2%)	141 (81.0%)
Abdominal pain	19 (10.2%)	18 (10.3%)
Chest pain	19 (10.2%)	19 (10.9%)
Fatigue	47 (25.3%)	43 (24.7%)
Fever	107 (57.5%)	99 (56.9%)
Myalgia	50 (26.9%)	47 (27.0%)
Nausea	21 (11.3%)	20 (11.5%)
Earache	8 (4.3%)	8 (4.6%)
Cough	119 (64.4%)	110 (63.2%)
Dizziness	2 (1.1.%)	2 (1.1%)
Vomiting	18 (9.7%)	18 (10.3%)
Oxygen support, *n* (%)		
No oxygen therapy	68 (36.6)	66 (37.9)
Oxygen therapy	104 (55.9)	98 (56.3)
Non‐invasive ventilation	14 (7.5)	10 (5.7)
Biochemical parameters		
Haemoglobin, mean (SD), g/L	12.6 (2.9) [*n* = 182]	12.7 (3.0) [*n* = 170]
Neutrophil, mean (SD), ×10^3^/mm^3^	6.6 (4.1) [*n* = 178]	6.5 (4.2) [*n* = 166]
Lymphocytes, mean (SD), ×10^3^/mm^3^	1.3 (1.1) [*n* = 176]	1.3 (1.2) [*n* = 165]
Platelets, mean (SD), ×10^3^/mm^3^	255.8 (126.1) [*n* = 181]	259.0 (128.6) [*n* = 169]
C‐reactive protein, mean (SD), mg/L	92.3 (87.7) [*n* = 167]	84.5 (80.6) [*n* = 156]
D‐dimer, mean (SD), ng/mL	2383.8 (4770.2) [*n* = 134]	2159.8 (3995.4) [*n* = 124]
Creatinine, mean (SD), mg/dL	1.4 (2.1) [*n* = 176]	1.2 (0.9) [*n* = 164]
Urea, mean (SD), mg/dL	51.8 (39.2) [*n* = 177]	48.6 (33.1) [*n* = 165]
Pulmonary commitment^a^ (≥50%), *n* (%)	42 (22.6%)	35 (18.8%)
ICU admission, *n* (%)	33 (17.7%)	22 (12.6)
Use of invasive mechanical ventilation, *n* (%)	7 (3.8)	1 (0.6%)
Hospital length of stay (day), median (IQR)	7 (4–11)	7 (4–11)
In‐hospital death, *n* (%)	12 (6.5%)	‐
Handgrip strength, kgF, median (IQR)	21 (15–30)	22 (15–30)
Vastus lateralis CSA, cm^3^, median (IQR)	12 (12–19)	16 (12–18)

BMI, body mass index; COPD, chronic obstructive pulmonary disease; CSA, cross‐sectional area; ICU, intensive care unit; IQR, interquartile range.

^a^ Pulmonary commitment was evaluated using chest computed tomography.

The signs and symptoms more commonly observed at admission were dyspnoea (81.2%), cough (64.4%), fever (57.5%), myalgia (26.9%), fatigue (25.3%), headache (22.0%), anosmia (19.9%), diarrhoea (17.7%), dysgeusia (17.2%), nausea (11.3%), abdomen pain (10.2%), chest pain (10.2%), vomiting (9.7%), runny nose (9.1%), earache (4.3%), and dizziness (1.1%). Mean (SD) LOS was 8.6 days (7.7); 17.0% of the patients required intensive care; 3.8% used invasive mechanical ventilation; and 6.6% died during the hospitalization period.

### Primary outcome

**Figure 1:**
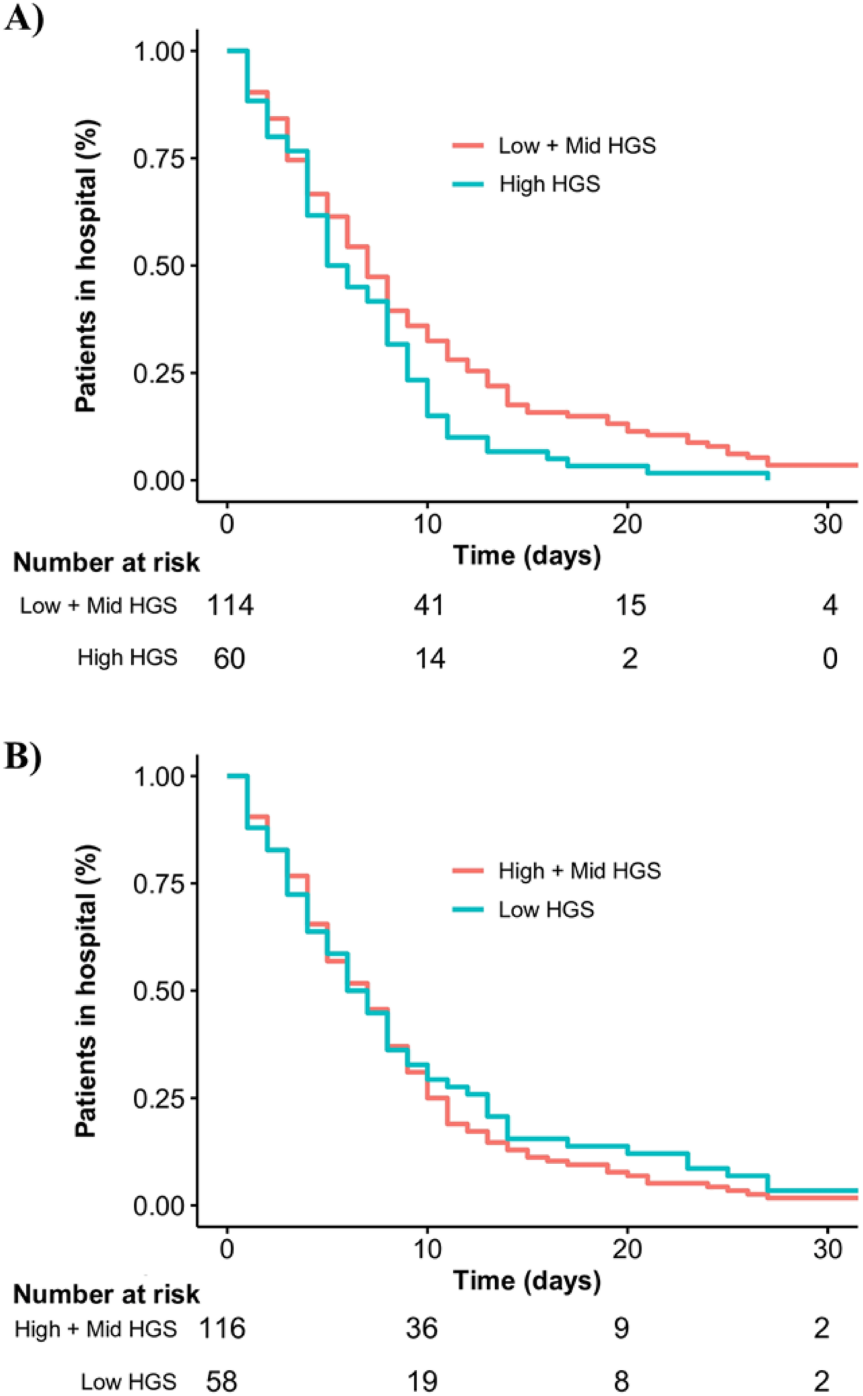
Kaplan–Meier plot of time from hospital admission to hospital discharge according to handgrip strength.

The crude HR for time from hospital admission to discharge was greatest for handgrip strength comparing the strongest versus other patients (1.47 [95% CI: 1.07–2.03; *P* = 0.019]). Evidence of an association between increased handgrip strength and shorter hospital stay was also identified when handgrip strength was standardized according to the sex‐specific mean and standard deviation (1.23 [95% CI: 1.06–1.43; *P* = 0.007). The magnitude of these associations remained consistent and statistically significant after adjusting for other covariates (*Figure* 1). Mean LOS was shorter for the strongest patients (7.5 ± 6.1 days) versus others (9.2 ± 8.4 days) (*Table* 2).

**Table 2:** Crude and adjusted hazard ratio (HR) for hospital length of stay in surviving patients

	Crude HR (95% CI)	*P* value	Adjusted HR (95% CI)	*P* value
Sex				
Male	1 (ref)		1 (ref)	
Female	0.82 (0.60–1.10)	0.186	0.81 (0.59–1.13)	0.216
Age, years				
18–35 years	1 (ref)		1 (ref)	
36–55 years	0.69 (0.37–1.29)	0.239	0.70 (0.36–1.37)	0.299
≥56 years	0.63 (0.35–1.15)	0.132	0.64 (0.34–1.20)	0.164
Oxygen support at admission				
0–4 L	1 (ref)		1 (ref)	
5–9 L	1.42 (0.92–2.19)	0.111	1.34 (0.85–2.11)	0.210
≥10 L	1.08 (0.63–1.84)	0.787	0.96 (0.54–1.72)	0.897
Obesity				
BMI < 30	1 (ref)		1 (ref)	
BMI ≥ 30	1.1 (0.80–1.47)	0.619	1.04 (0.66–1.30)	0.802
Type 2 diabetes				
Yes	1 (ref)		1 (ref)	
No	0.93 (0.67–1.28)	0.637	0.93 (0.66–1.30)	0.667
Handgrip strength: High vs. Other				
Other	1 (ref)		1 (ref)	
High	1.47 (1.07–2.03)	0.019*	1.48 (1.05–2.09)	0.024*
CSA_VL_: High vs. Other				
Other	1 (ref)		1 (ref)	
High	1.05 (0.76–1.45)	0.770	0.88 (0.59–1.32)	0.534
Handgrip strength: Low vs. Other				
Other	1 (ref)		1 (ref)	
Low	0.90 (0.65–1.24)	0.510	0.94 (0.66–1.33)	0.713
CSA_VL_: Low vs. Other				
Other	1 (ref)		1 (ref)	
Low	0.63 (0.46–0.88)	0.006**	0.59 (0.40–0.87)	0.007**
Handgrip strength: Standardized	1.23 (1.06–1.43)	0.007**	1.26 (1.07–1.48)	0.005**
CSA_VL_: Standardized	1.20 (1.03–1.39)	0.016*	1.24 (1.03–1.50)	0.023*

CSA_VL_, vastus lateralis cross‐sectional area.

The Cox proportional hazards model was adjusted by sex (male or female), age (18–35, 36–55, or ≥56 years), oxygen support at admission (0–4 L, 5–10 L, and ≥10 L), obesity (BMI < 30 and BMI > 30), and type 2 diabetes (yes or no).

* *P* < 0.05.

** *P* < 0.01.

**Figure 2:**
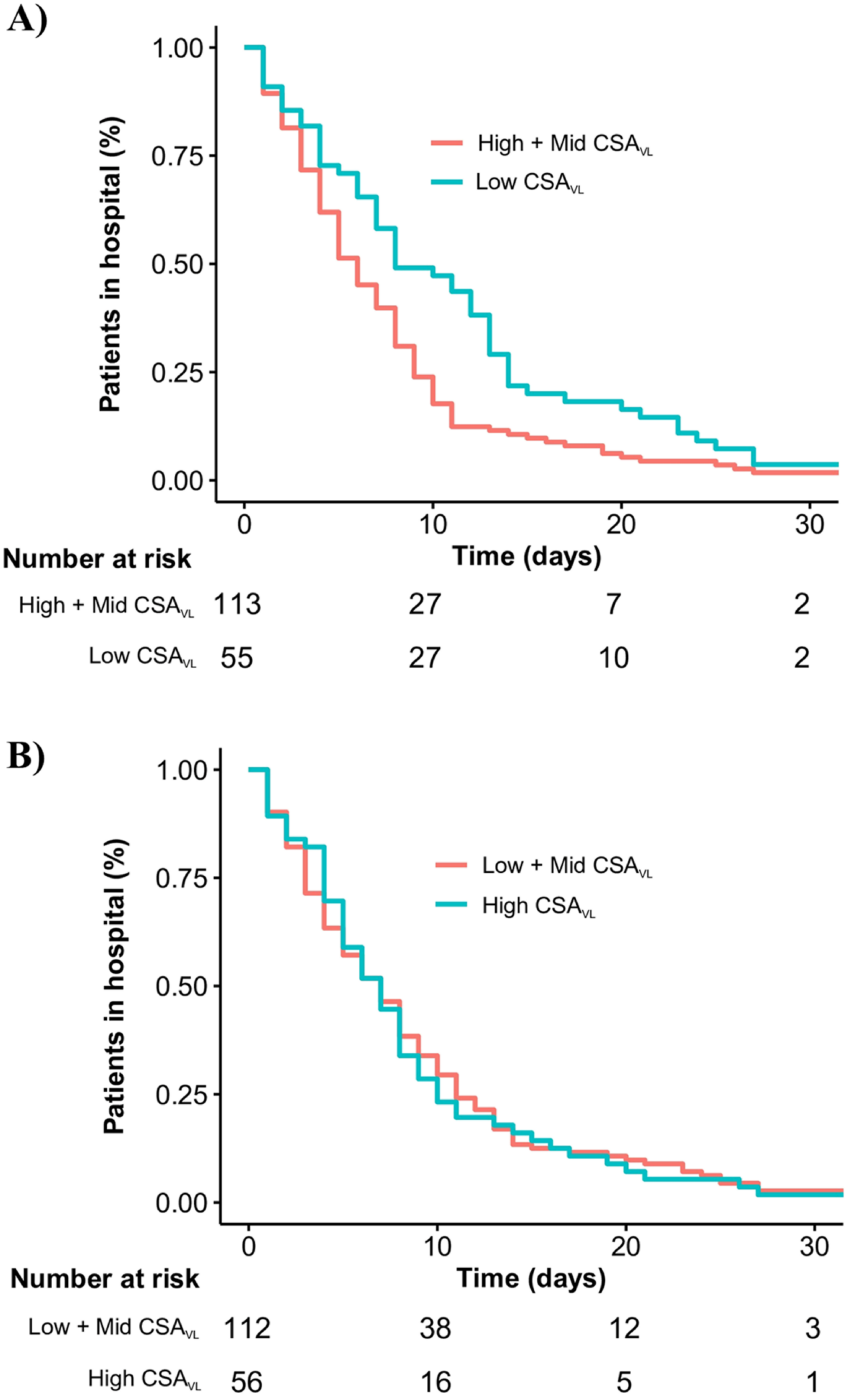
Kaplan–Meier plot of time from hospital admission to hospital discharge according to vastus lateralis cross‐sectional area.

Similar results and evidence of associations were also present for vastus lateralis cross‐sectional area. The crude HR identified shorter hospital stay for patients with greater sex‐specific standardized values (1.20 [95% CI: 1.03–1.39; *P* = 0.016). Evidence was also obtained associating longer hospital stays for patients with the lowest values for vastus lateralis cross‐sectional area (0.63 [95% CI: 0.46–0.88; *P* = 0.006). The magnitude of these associations remained consistent and statistically significant after adjusting for other covariates (*Figure* 2). Mean LOS for the patients with the lowest muscle cross‐sectional area was longer (10.8 ± 8.8 days) versus others (7.7 ± 7.2 days) (*Table* 2).

## Discussion

In this prospective observational study, we found muscle strength (as assessed by handgrip) and muscle mass (as assessed by vastus lateralis cross‐sectional area) are predictive of LOS in hospitalized patients with moderate to severe COVID‐19. To the best of our knowledge, this is the first study to demonstrate the prognostic value of these skeletal muscle parameters in this disease.

A recent study demonstrated that the Clinical Frailty Score (CFS) independently predicted time to medical discharge and mortality in COVID‐19 patients.^20^ Despite the value of these findings, it is noteworthy that the CFS is a judgement‐based frailty tool that relies highly on experience and training for proper categorization of the patients. Moreover, CFS is ultimately an indirect measure of functional status and is mainly used in geriatric patients. These are factors that might limit the reliability of CFS in real‐life clinical scenarios. Conversely, handgrip strength is a simple, direct, easy handling, low‐cost measurement commonly utilized in the clinical setting as an indicator of the general health status in individuals across a wide age range. Indeed, handgrip strength assessed at hospital admission have been shown to be a predictive measure of LOS and mortality in distinct populations.^7^, ^8^, ^9^, ^10^, ^11^ Our findings extend this knowledge to patients admitted into the hospital with acute COVID‐19 symptoms, by showing that stronger patients had lower LOS than their weaker counterparts.

Muscle mass is also considered as an indicator of general health status.^24^, ^25^ Previous studies have suggested that low muscle mass (assessed by mid‐arm circumference, calf circumference, and estimated by anthropometric equations) may predict mortality among elderly.^24^, ^25^ In the current study, we directly assessed, in the point‐of‐care, vastus lateralis cross‐sectional area using ultrasonography among patients with COVID‐19. Our findings suggest that low muscle mass could contribute to higher LOS among COVID‐19 patients. During a critical illness, net breakdown of muscle protein is stimulated to provide abundant amino acids to meet these increased demands of tissues such as immune cells and liver.^14^ In this context, patients with limited muscle mass reserves would presumably be more vulnerable to stress factors, such as severe burn injuries and cancer.^26^, ^27^ The present findings suggest that this could be the case of COVID‐19.

Muscle mass plays a key role in recovery from critical illness, whereas muscle strength and function are key to the recovery process.^12^ If there is a preexisting deficiency of muscle mass before the onset of an acute illness, one may speculate that the expected loss of muscle mass and function associated with hospitalization may push the patient over a threshold that makes recovery of normal function unlikely to ever occur.^12^ The impact of this physiopathological mechanism on long‐term effects of COVID‐19 (long COVID) remains to be explored.

### Limitations

First, the longitudinal design of this study does not allow causative conclusions. Second, although this study was adequately powered to detect changes in the selected outcomes, this was still a small cohort composed by patients with heterogeneous clinical features, medication regimen and disease manifestations, possibly subject to unmeasured confounders. While the Cox proportional hazards models were controlled for several potentially confound variables, direct sub‐group comparisons were not possible due to sample size constraints. Third, our results are confined to patients with moderate to severe COVID‐19 and should be read with care regarding other clinical settings. Finally, the minimal clinically important difference in LOS among patient with COVID‐19 is yet unknown, which limits the ability to make clinical inferences about the present findings.

## Conclusions

Muscle strength and mass assessed on hospital admission are predictors of LOS in patients with COVID‐19. While it is unknown whether these muscular parameters add to the prognostic value provided by the more established and accepted predictors that already have been identified,^28^ the present data suggest that muscle health may benefit patients with moderate to severe COVID‐19. The evidence provided by this study paves the way for randomized controlled trials to test the utility of preventive or in‐hospital interventions in shortening LOS among these patients through improving muscle mass and/or function.

## Conflict of interest

The authors have declared that no conflict of interest exists.

## Funding

The authors acknowledge the support by Coordenação de Aperfeiçoamento de Pessoal de Nível Superior (CAPES ‐ PROEX), the Brazilian National Council for Scientific and Technological Development (CNPq, grant 301571/2017‐1). S.G., H.R., and B.G. are supported by grants from the Conselho Nacional de Pesquisa e Desenvolvimento (CNPq, 166622/2020‐6; 428242/2018‐9; and 301914/2017‐6). B.G. and S.G. is also supported by a grant from the Sao Paulo Research Foundation (FAPESP 2017/13552‐2; 2020/08091‐9).
